# Case Mix and Protocol Constraints in Diagnostic Language Model Conversations: A Secondary Analysis of Released MedR-Bench Outputs

**DOI:** 10.7759/cureus.118344

**Published:** 2026-10-21

**Authors:** Oscar Mingtian Li, Summer Xia Tian Li, Yijiang Li

**Affiliations:** 1 Zweistein Innovation Lab, Active Care and Education in Sports and Outreach Foundation (ACESO Foundation), Dublin, USA

**Keywords:** benchmark evaluation, cardiovascular informatics, clinical decision support, diagnostic reasoning, large language models, reproducibility, simulator feedback

## Abstract

Introduction

Diagnostic language models can request additional examinations before answering. Their observed stopping behavior depends on the model, the information returned by a simulator, and the interaction protocol, and these contributions are difficult to separate in aggregate benchmark results. We quantified an explicit unavailable-information text marker in simulator feedback and its association with fifth-response contact, a protocol-imposed interaction limit.

Methods

We analyzed released MedR-Bench diagnosis conversations from seven model labels and 957 source case reports. Exposure was a text marker: the exact simulator sentence stating that no relevant ancillary test information was available, in the first simulator feedback. It was not a clinical judgment that information was missing. The outcome was fifth-response contact, meaning that the run reached the fifth assistant response. Within-model risk differences were averaged with equal model weights, with 5,000 report-cluster bootstrap resamples. Sensitivity analyses used linear probability models with model effects and then report and model effects, with and without first-request composition, and leave-one-model-out summaries.

Results

Of 6,699 expected report-model cells, 6,690 were released, and 5,719 entered the first-feedback analysis; entry ranged from 41.2% to 98.0% of released runs by model. The marker appeared in 4,593 first feedbacks (80.3%), and 96.2% of these also returned other text for at least one requested item. All 1,001 fifth-response runs followed a mandatory-final-answer prompt. The equal-model-weighted difference was 6.37 percentage points (95% CI 4.16 to 8.54), or 4.16 points without the model with the largest difference. The difference largely reflected case composition: the common-slope coefficient was 5.61 points with model effects, 1.05 points (95% CI -1.64 to 3.74) with report and model effects, and 1.21 points (-1.67 to 4.08) after adding request composition. In paired one-round and free-turn releases, marker status differed in 1,309 of 5,701 pairs (23.0%) despite identical case text.

Conclusions

The positive association between the unavailable-information text marker and fifth-response contact was largely explained by case composition and was close to null within source reports. Case composition and protocol states should remain visible when diagnostic trajectories are evaluated. These data do not establish a causal effect of missing information or support heart-failure clinical validation.

## Introduction

A diagnostic conversation is shaped by what information can be obtained. A model may request an examination that is absent from a published case report, receive a reply that no relevant result is available, and then ask again, change its request, or answer. Those actions are observable. Whether the examination was necessary, whether it had been performed in clinical care, and whether stopping was safe are different questions.

Recent benchmarks have moved beyond a final-answer score. MedR-Bench evaluates examination requests and diagnostic reasoning in conversations derived from published cases [1]. Sequential Diagnosis and AgentClinic likewise study interactive information acquisition [2,3]. CliniCARE-Bench examines evidence grounding and process requirements in electronic health record tasks, while CardioBench evaluates cardiovascular language-model tasks [4,5]. These studies establish the importance of process evaluation. They also leave a practical measurement question: how should a released trajectory be interpreted when the simulator cannot supply a requested result or when the protocol requires the conversation to end?

This is a methodological study of benchmark evaluation, and its lessons are not specific to one specialty. We use cardiovascular medicine as the applicability example because cardiovascular decision support is a common intended use of diagnostic language models and because the benchmark provides a source-defined cardiovascular category. The question matters for cardiovascular artificial intelligence (AI) development because a plausible process metric can easily acquire a clinical interpretation that its data do not support. A blood and circulation category is broader than heart failure. An unavailable-result message is not an adjudicated clinical omission. A final answer after a mandatory stop prompt does not reveal when the model would have stopped without that prompt. Work on benchmark integrity has similarly emphasized the need to separate defects in an evaluation setting from the behavior attributed to a model [6].

We analyzed the complete released MedR-Bench free-turn diagnosis file. The primary aim was to estimate whether an explicitly unavailable-information text marker in the first simulator feedback was associated with fifth-response contact, that is, with the conversation reaching the fifth assistant response. We used the first feedback to avoid defining exposure by events that become more likely simply because a conversation lasts longer. Two secondary aims served this question. The first was applicability: to assess the source-defined blood, heart, and circulation subset and to examine how much heart-failure-specific material could actually be identified, because a cardiovascular reading of these data is the most likely over-interpretation. The second was protocol dependence: to examine whether the observable states used in the primary analysis remain stable when the interaction protocol changes, first in the paired MedR-Bench one-round release and then, as a separate check in a different benchmark with a different endpoint, in CARE-Bench Public Test 1. The secondary analyses are reported separately and are not combined with the primary estimate. The study evaluates observable benchmark behavior; it does not validate a clinical scoring system or compare diagnostic accuracy.

## Materials and methods

Design and source artifacts

This was a post hoc observational analysis of previously released model outputs. The questions and extraction rules were developed after inspecting the source code, corpus structure, and an earlier internal structural audit of the cardiovascular subset by the authors. That audit was an unpublished internal working analysis and has not been released as a preprint or other citable record. It informed which message fields and text patterns were extracted, and it is the source of the permissive stop-phrase rule compared in the lexical diagnostics; none of its estimates is reported here. The analysis was not preregistered. No new model inference, automated clinical judging, or clinical expert labeling was performed. Reporting was informed by the applicable items of the Transparent Reporting of a multivariable prediction model for Individual Prognosis Or Diagnosis (TRIPOD)-LLM reporting guideline for studies using large language models [7]; a completed reporting checklist is not treated as evidence of clinical validity. Because the unit of analysis is released benchmark output rather than a body of published studies, this is not a systematic review, and systematic-review reporting guidelines do not apply.

The study used only publicly released, de-identified derivatives of published case reports and previously generated model outputs. It involved no contact with human participants, no new clinical intervention, no access to restricted electronic health records, and no linkage of patient identities. Institutional review board approval and informed consent were, therefore, not required.

The diagnosis file contained 957 source-report keys. We separately inspected 496 treatment reports to assess data coverage, but the released output file contained diagnosis conversations only. The source repository was analyzed at commit ff60ab440afd2f2bc0c603b3a65d715ec83138a7 [8]. The inference file came from the separately released output dataset [9]; it was not tracked by that code commit, and its SHA-256 checksum (08fe35b265f029219768b459ed7348a2ea60a6274adba3d93b786e1fdc5e24aa) fixes the analyzed bytes. The public inference script differs from the released payload in field names and model dispatch and was not treated as a reconstruction of the original generation environment. Protocol states were, therefore, verified from the actual saved messages.

Seven model labels were present: Baichuan-M1, DeepSeek-R1, DiagnoseGPT, Gemini2-FT, MedGemma, o3-mini, and QwQ. We retained these release labels rather than inferring current product versions or rerunning the models. Exact deployment revisions, sampling configuration, and run-level seeds could not all be reconstructed from the released payload. Consequently, the findings refer to these saved runs. The developer and documented version for each label are listed under the section titled "Software, models, and computational tools".

Units and complete denominators

The analysis hierarchy was source report, diagnostic item, model run, and ordered message. A PubMed Central (PMC) identifier denotes a source report; it does not establish a unique patient. All seven expected model cells were retained for every report, including absent outputs. Available runs were then divided into those with one assistant response and no simulator feedback, and those with at least one feedback followed by another assistant response. Only the latter entered the first-feedback analysis. Runs outside that analysis remain visible in the overall denominator.

Source-report identifiers were the resampling units. All model runs for a sampled report moved together in each bootstrap replicate. We did not link patient identities across reports or datasets. Reusing a report across models increases the number of model observations, not the number of independent people.

Feedback and protocol outcomes

We extracted each simulator feedback body between the released "Additional Information" prefix and the following response-format instructions. Searching the whole user message would risk counting instructional text as returned evidence. Every one of the 10,362 feedback events had one recognized prefix and suffix. The binary exposure was the case-insensitive occurrence of the standard sentence "There is no relevant ancillary test information available for this request." in the first extracted feedback body. Absence of this exact sentence was not interpreted as complete information: the simulator could use another expression or return a partial result.

Some marker-positive feedback also contained examination names, list formatting, or other text. We, therefore, distinguished normalized single-sentence-only feedback from marker-positive feedback with additional text. The latter was not classified as containing useful clinical evidence without further adjudication. Conversely, a marker about one requested examination was not interpreted as meaning that every requested result was unavailable.

In a post hoc analysis added at peer review, we decomposed each marker-positive first feedback by requested item. The extracted body was split at its top-level numbered or bulleted items, and an item was counted as returning other content when, after removal of the standard sentence, the item label and formatting characters, it still contained more than 15 letters or digits. Marker-positive feedback was classified as marker-only when no item returned other content and as mixed otherwise. This is a lexical rule: it shows whether the simulator returned any other text alongside the marker, not whether that text was clinically useful, and no clinical adjudication was performed. The rule was checked by reading random samples of feedback bodies from both classes.

The main outcome was fifth-response contact, defined as the run reaching the fifth assistant response. We separately extracted the protocol instruction requiring a final diagnosis without additional questions on the next turn and checked its position. This instruction appears before the fifth response. Earlier endings were described by their observed position; we did not infer that they reflected adequate evidence or clinically appropriate decisions. The outcome measures a protocol state rather than diagnostic success or failure.

Statistical analysis

Within each model, we calculated the proportion of eligible runs reaching the fifth response in the marker-positive and marker-negative groups. The within-model risk difference was the first proportion minus the second. The principal summary was the unweighted mean of the seven differences. This gives each released model label the same weight, even though their frequencies of entering the first-feedback analysis differ. Its two component proportions are standardized descriptive averages, not raw pooled proportions.

We used 5,000 nonparametric source-report bootstrap samples with seed 20260917 and percentile 95% intervals. Each full-corpus resample contained 957 report draws. Model-specific contrasts were reported descriptively, without selecting a best model or testing a family of superiority hypotheses. The principal contrast does not adjust for case difficulty, request composition, or simulator response behavior. It, therefore, estimates an association among runs that entered the first-feedback sample, not the effect of withholding a result.

We additionally fitted common-slope linear probability models, first with released-model indicators and then with source-report and model indicators. The second model uses within-report contrasts after accounting for average model differences. Coefficients were estimated by residualizing exposure and outcome against the fixed effects, and a separate Frisch-Waugh-Lovell calculation checked the result. Confidence intervals used a source-report cluster sandwich variance with the CR1 finite-sample adjustment and normal quantiles. The regression coefficients have different weights from the equal-model summary; we, therefore, compare the two regression specifications with each other. These post hoc sensitivities cannot remove model-specific request selection or simulator confounding.

Further post hoc sensitivity analyses were added at peer review. First, to show how much within-report contrast identified the report-adjusted coefficient, we counted the source reports that contributed both marker-positive and marker-negative eligible runs. Second, we added observable characteristics of the first information request to the report-and-model regression: the number of requested items (one, two, three, four, five, or six or more, counted from the top-level numbered or bulleted lines of the first request section) and six non-exclusive lexical indicators for laboratory or pathology tests, imaging, physical examination, history or symptoms, genetic testing, and other investigations such as electrocardiography or endoscopy. Third, we recomputed the equal-model-weighted summary seven times, each time omitting one model label, with 5,000 report-cluster bootstrap resamples. Fourth, we refitted both regression specifications with separate indicators for marker-only and mixed first feedback, with marker-negative feedback as the reference. These analyses were specified after the reviewers' comments and are exploratory.

The nested blood, heart and circulation analysis used the same extraction and contrast definitions. Sparse marker-negative cells were assessed explicitly. Where resamples lacked an exposure group, the resulting conditional interval was retained only as a diagnostic and was not presented as an ordinary confidence interval. Heart-failure screens were descriptive only. A wording sensitivity analysis permitted variable whitespace, an optional additional adjective, and punctuation differences in the marker sentence, and a complete-grid sensitivity analysis restricted the sample to reports with all seven released outputs.

Cardiovascular applicability and lexical diagnostics

The cardiovascular metadata subset used exact membership of "Blood, Heart and Circulation" in the body_category field. This broad category includes material beyond heart failure and should not be interpreted as an adult heart-failure cohort.

For feasibility, a case-insensitive word-boundary rule searched for heart failure, cardiac failure, heart failure with reduced ejection fraction (HFrEF), heart failure with preserved ejection fraction (HFpEF), or heart failure with mildly reduced ejection fraction (HFmrEF). We applied it separately to the generated diagnosis-results field, the generated final-diagnosis field, the case summary, and any source field. These searches were deliberately kept separate. They can detect mentions in background, differential diagnoses, or negated statements; they do not adjudicate disease status.

A subsequent targeted source-context audit screened species and veterinary terms, then checked the subject identified in the corresponding released report fields. This was a search for explicit counterexamples to a human-cohort interpretation, not exhaustive species adjudication. The primary analysis retained the entire release; excluding the explicitly nonhuman reports was an additional point-estimate sensitivity.

Secondary lexical diagnostics counted repeated entire information-request blocks before and after removing list markers, punctuation, case, and redundant whitespace. These are reproducibility measures. An exact repeat need not be clinically redundant, and a rephrased repeat can be missed. We also compared canonical terminal stop phrases with a more permissive substring rule used in the earlier audit. No lexical measure was used as a clinical reference standard.

Paired protocol extensions

After the main analysis, we examined two additional MedR-Bench releases: one information round followed by a final response, and an oracle condition supplying the recorded case information [9]. The one-round condition contains two assistant responses, not one. We constructed a three-condition report-model grid and compared hashes of the saved system instructions, initial user messages, extracted source-case sections, first assistant answers, and first-feedback bodies. In pairs with feedback under both one-round and free-turn conditions, we estimated the free-turn-minus-one-round marker-rate difference within each model and then averaged the seven differences equally, with 5,000 source-report cluster bootstrap resamples (seed 20260922). This was an additional post hoc analysis; protocol, prompt, and generated-feedback differences were not randomized or experimentally isolated. The one-round pipeline requests simulator feedback even when the first assistant response declines further information, whereas the free-turn pipeline can stop without feedback. Requiring feedback under both conditions, therefore selects a conditional comparison set. To describe how the first requests themselves differed, we compared within each pair the number of requested items and the set of lexical request categories defined above in the first assistant response. Because the full prompts and first requests differ between releases, the paired comparisons are presented as descriptive differences between released protocol conditions and not as an isolated effect of the turn budget.

As a separate secondary analysis, we asked whether protocol dependence of a trajectory summary also appears in a different benchmark with a different endpoint. For this, we examined the author-released mapped predictions for CARE-Bench Public Test 1: 11 model labels, prompted and unprompted protocols, 135 patient-disclosure prefixes, and 62 cases [10]. Each prefix was submitted separately as cumulative patient information, without earlier assistant responses, so these records are not complete interactive rollouts. CARE-Bench already reports trajectory exact match, defined as agreement with its action reference at every evaluated prefix of a case. We reproduced that endpoint and added a paired decomposition into agreement under both protocols, prompted only, unprompted only, or neither. The overall difference gave equal weight to each case and model; 5,000 whole-case bootstrap resamples (seed 20260921) retained all prefixes, models, and protocols within each case. The released labels are benchmark reference and mapping artifacts; raw model responses were unavailable, so we could not separate changes in recommendations from changes introduced by response-to-label mapping. These analyses were kept separate from the MedR-Bench endpoints. No new clinical reference was created, and no generative model was trained or rerun.

Software, models, and computational tools

All analyses were performed in Python version 3.12.10 (Python Software Foundation, Wilmington, DE, USA). Data handling, bootstrap resampling, and the linear probability models used NumPy version 2.4.6 (NumPy Developers; fiscally sponsored by NumFOCUS, Austin, TX, USA). SHA-256 checksums were computed with the hashlib module of the Python 3.12.10 standard library. Figures were drawn directly from the numerical results with Matplotlib version 3.10.9 (Matplotlib Development Team; fiscally sponsored by NumFOCUS, Austin, TX, USA). No commercial statistical package was used, and the authors did not run, fine-tune, or query any language model for this analysis. The additional analyses requested at peer review were run in October 2026 in JavaScript in the Google Chrome browser, version 155 (Google LLC, Mountain View, CA, USA), on the same release file, verified by its SHA-256 checksum. Before any new estimate was computed, this re-implementation reproduced every original first-feedback count, the seven model-specific differences, and both full-corpus regression coefficients and their cluster-robust intervals to two decimal places. Its bootstrap used a different random-number generator (xorshift, seed 20261014), so its intervals are not draw-for-draw identical to those obtained with NumPy.

The saved MedR-Bench conversations were generated by the benchmark developers. The model behind each release label, with developer and version as documented in the benchmark publication and code at the analyzed commit [1,8], was: Baichuan-M1, Baichuan-M1-14B-Instruct (Baichuan Intelligent Technology, Beijing, China); DeepSeek-R1, DeepSeek-R1 671B (DeepSeek, Hangzhou, China); DiagnoseGPT, DiagnosisGPT-34B (FreedomIntelligence, The Chinese University of Hong Kong, Shenzhen, China); Gemini2-FT, gemini-2.0-flash-thinking-exp-01-21 (Google LLC, Mountain View, CA, USA); MedGemma, the 27-billion-parameter Gemma 3-based MedGemma model (Google LLC, Mountain View, CA, USA), for which the released code does not name a checkpoint; o3-mini, o3-mini-2025-01-31 (OpenAI, San Francisco, CA, USA); and QwQ, QwQ-32B-Preview (Alibaba Group, Hangzhou, China). In the released code, the simulator that returned requested examination results was GPT-4o, gpt-4o-2024-11-20 (OpenAI, San Francisco, CA, USA). Because the public inference script is not a reconstruction of the original generation environment, these are the documented versions rather than versions verified from the saved outputs.

The CARE-Bench mapped predictions [10] were generated by the benchmark developers with the following models, as recorded in the benchmark's evaluated-model registry at the analyzed commit: Claude Haiku 4.5, claude-haiku-4-5-20251001, and Claude Opus 4.7, claude-opus-4-7 (Anthropic, San Francisco, CA, USA); Gemini 3.1 Flash Lite, gemini-3.1-flash-lite, Gemini 3.1 Pro, gemini-3.1-pro-preview, and MedGemma 27B, medgemma-27b-text-it (Google LLC, Mountain View, CA, USA); GPT-5.4 mini, gpt-5.4-mini-2026-03-17, and GPT-5.5, gpt-5.5-2026-04-23 (OpenAI, San Francisco, CA, USA); HuatuoGPT-o1, HuatuoGPT-o1-8B (FreedomIntelligence, The Chinese University of Hong Kong, Shenzhen, China); Llama 4 Scout, llama-4-scout-17b-16e-instruct (Meta Platforms, Menlo Park, CA, USA); Mistral Large 3, mistral-large-2512 (Mistral AI, Paris, France); and Qwen3-32B (Alibaba Group, Hangzhou, China). Free-text responses were mapped to the four action labels by the developers with GPT-5.5, gpt-5.5-2026-04-23 (OpenAI, San Francisco, CA, USA).

Use of AI assistance

OpenAI Codex (OpenAI, San Francisco, California, USA), accessed through the Codex application during 2026, was used to assist literature retrieval, analysis code development, drafting, and internal methodological review; the underlying model version was not recorded. Claude Opus 5 (model identifier claude-opus-5; Anthropic, San Francisco, CA, USA), accessed through the Claude desktop application, was used on 17 and 18 September 2026 to reformat the manuscript for journal style, verify reference metadata against publisher and Crossref records, compile the software and model version details above from the benchmark publications, code and model registry, and edit the Matplotlib plotting script so that the figures carry no in-image titles. Claude Opus 5.5 (model identifier claude-opus-5-5; Anthropic, San Francisco, CA, USA) was used on 14 October 2026 to write and run the JavaScript re-implementation and the peer-review sensitivity analyses described above, to check the item-level feedback rule against random samples of feedback text, and to draft the revised text and the responses to reviewers. Both figures are Matplotlib renderings of the numerical results. All numerical results were checked against the analysis outputs by the authors, who take full responsibility for the content.

## Results

Release completeness and observed protocol states

The complete expected grid contained 6,699 report-model cells. Of these, 6,690 were released (99.87%), and nine were absent: four for DiagnoseGPT, four for QwQ, and one for MedGemma. All released conversations followed the expected system-user-assistant alternation and contained between one and five assistant responses.

There were 971 runs with no simulator feedback (14.5% of released runs) and 5,719 eligible runs with at least one piece of feedback. Entry into the latter group varied considerably: Baichuan-M1 contributed 938 eligible runs from 957 released outputs (98.0%), QwQ 930 of 953 (97.6%), Gemini2-FT 918 of 957 (95.9%), o3-mini 902 of 957 (94.3%), DeepSeek-R1 871 of 957 (91.0%), MedGemma 767 of 956 (80.2%), and DiagnoseGPT 393 of 953 (41.2%). Each model-specific contrast below therefore describes a differently selected subset of cases; for DiagnoseGPT, it concerns the minority of cases in which the model requested more information at all. These selection differences are part of the behavior being described, and they prevent a simple all-run model ranking from the subsequent conditional contrast.

In total, 1,001 runs reached the fifth assistant response, representing 15.0% of released runs and 17.5% of eligible runs. Every such run contained the mandatory-final-answer instruction, and that instruction did not occur in any run ending before the fifth response. Figure 1 shows the intended denominator, no-feedback state, earlier endings, and contact with the protocol limit by model.

**Figure 1:**
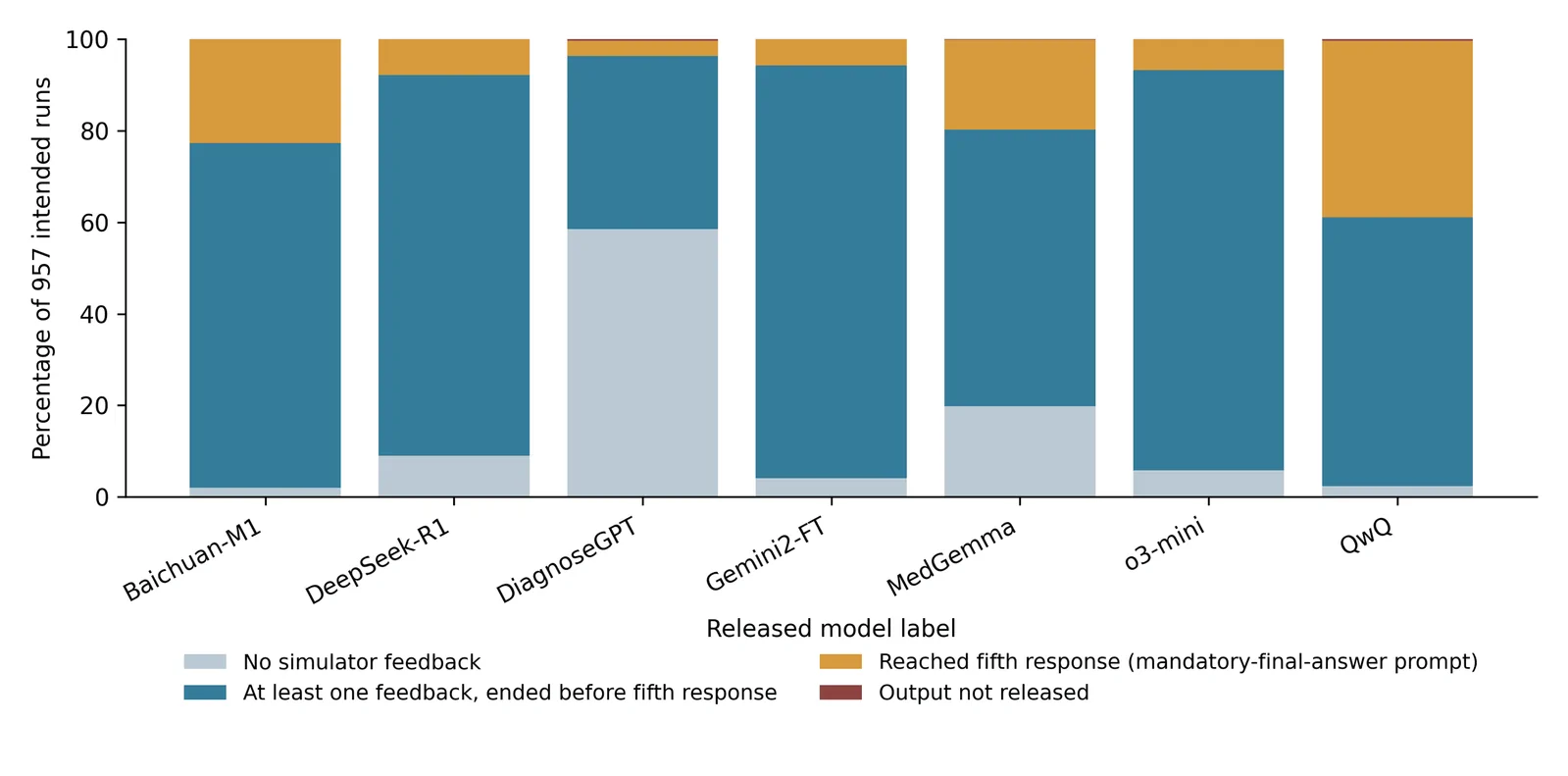
Intended denominators and observed protocol states by released model label Stacked bars show, for each of the seven released model labels (horizontal axis), the percentage of its 957 intended report-model runs (vertical axis) in each state: no simulator feedback (light grey), at least one simulator feedback followed by an ending before the fifth assistant response (blue), reaching the fifth assistant response (orange), and output not released (dark red; nine runs overall). Every run that reached the fifth response had received the protocol's mandatory-final-answer instruction, and no run ending earlier had received it. Absent releases are kept in the denominator.

First feedback and fifth-response contact

The exact unavailable-information marker occurred in 4,593 of 5,719 first feedback (80.3%). After normalization, 27 consisted solely of the standard sentence; 4,566 contained that sentence together with additional text. Item-level decomposition showed that the marker rarely meant that nothing was returned: in 4,420 marker-positive first feedback (96.2%), at least one requested item was answered with other text, and only 173 (3.8%) were marker-only across every requested item. Whether the other text was clinically informative was not adjudicated. Across all 10,362 feedback events, 8,565 contained the marker; this event-level count is descriptive because later feedback opportunities depend on conversation length.

Table 1 presents the first-feedback comparison. Equal-model-weighted fifth-response proportions were 17.71% after marker-positive feedback and 11.34% after marker-negative feedback. The difference was 6.37 percentage points (95% report-cluster bootstrap interval 4.16 to 8.54). All 5,000 full-corpus resamples yielded an estimable seven-model contrast.

**Table 1:** First-feedback unavailable-information marker and reaching the fifth assistant response, by released model label Each row gives, for one released model label, the number of first-feedback eligible runs, then for runs whose first simulator feedback did and did not contain the exact unavailable-information sentence the number that reached the fifth assistant response, the number of runs, and the percentage. The difference is the marker-present percentage minus the marker-absent percentage, in percentage points. The 95% confidence interval (CI) limits are percentile limits from 5,000 bootstrap resamples of source reports (seed 20260917), in which all model runs for a report move together. In the summary row, the two percentages are the unweighted means of the seven model-specific percentages and the difference is the unweighted mean of the seven model-specific differences, so counts are not pooled. All contrasts condition on entering the first-feedback analysis. A marker indicates the exact simulator sentence and does not establish that no information was returned. No clinical reference standard was used.

Released model label	Eligible runs	Marker present: reached fifth response, n	Marker present: runs, n	Marker present: reached fifth response, %	Marker absent: reached fifth response, n	Marker absent: runs, n	Marker absent: reached fifth response, %	Difference, percentage points	95% CI lower limit	95% CI upper limit
Baichuan-M1	938	138	547	25.2	80	391	20.5	4.77	-0.75	10.13
DeepSeek-R1	871	69	743	9.3	6	128	4.7	4.60	0.15	8.46
DiagnoseGPT	393	23	280	8.2	8	113	7.1	1.13	-4.83	6.67
Gemini2-FT	918	54	790	6.8	1	128	0.8	6.05	3.66	8.19
MedGemma	767	157	622	25.2	31	145	21.4	3.86	-3.92	11.34
o3-mini	902	60	755	7.9	5	147	3.4	4.55	0.86	7.88
QwQ	930	353	856	41.2	16	74	21.6	19.62	9.51	29.27
Equal-model-weighted summary	5719	Not pooled	Not pooled	17.71	Not pooled	Not pooled	11.34	6.37	4.16	8.54

The within-model point differences ranged from 1.13 percentage points for DiagnoseGPT to 19.62 for QwQ (Figure 2). Several model-specific intervals included zero. These differences describe saved trajectories and do not identify a model's response to a randomized change in available information.

**Figure 2:**
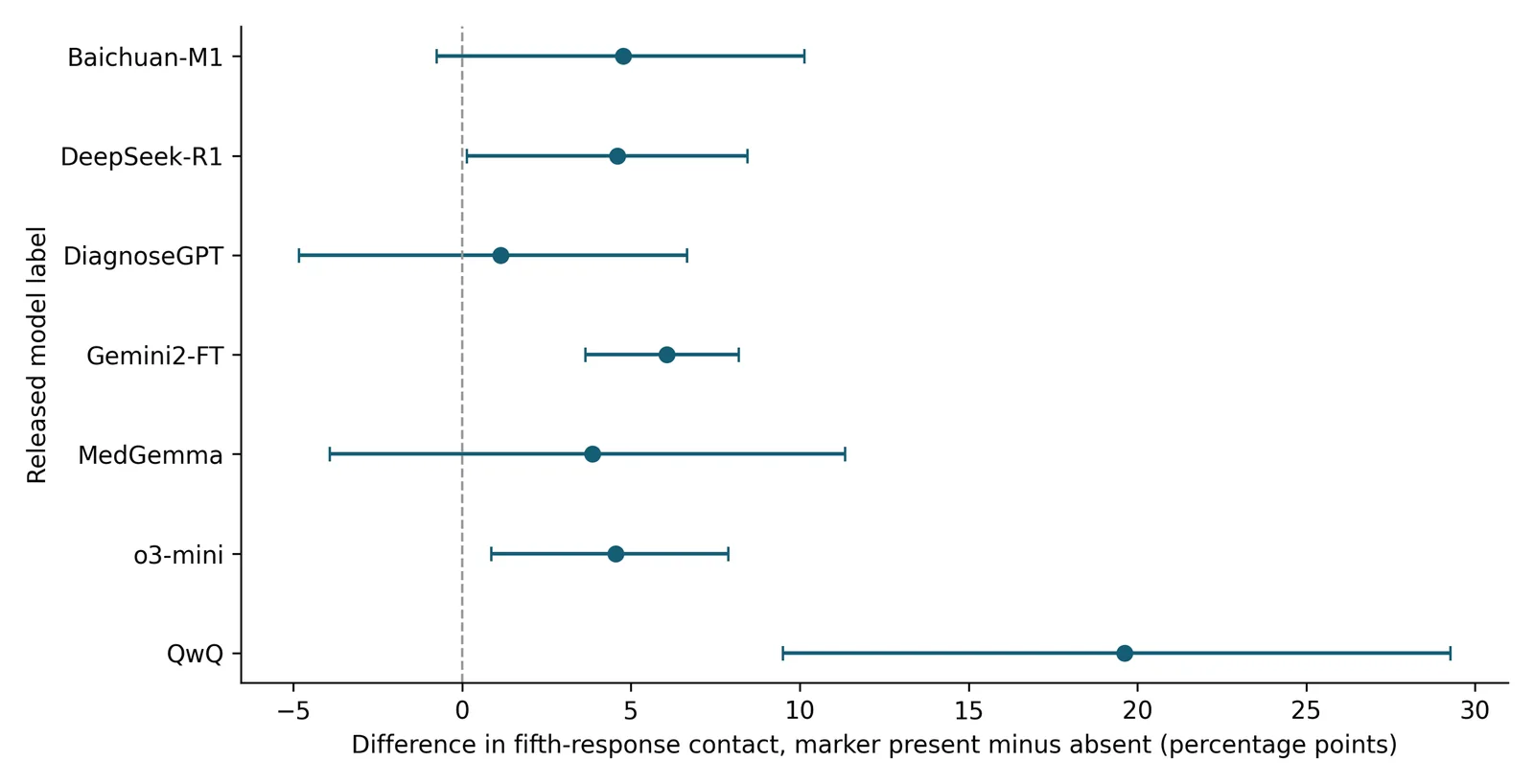
Within-model difference in reaching the fifth response by first-feedback marker status Points show, for each released model label (vertical axis), the marker-present minus marker-absent difference in the percentage of first-feedback eligible runs that reached the fifth assistant response, in percentage points (horizontal axis); horizontal bars are 95% percentile intervals from 5,000 source-report bootstrap resamples. The dashed vertical line marks no difference. Models enter the first-feedback sample at different rates, so the figure is not a ranking of clinical quality.

Omitting one model label at a time, the equal-model-weighted difference ranged from 6.42 to 7.24 percentage points for six of the seven omissions. Omitting QwQ reduced it to 4.16 percentage points (95% report-cluster bootstrap interval 2.11 to 6.20), so the seven-model summary is partly driven by that single label.

Sensitivity to report composition and extraction

On the same 5,719 eligible runs from 954 source reports, the model-only linear probability coefficient was 5.61 percentage points (95% cluster-robust interval 3.41 to 7.82). After adding source-report effects, it was 1.05 points (-1.64 to 3.74). The common-slope coefficient was substantially smaller after adding report indicators. This is not proof of a zero association: the interval includes both modest negative and positive values, and request-specific confounding remains. Table 2 places the distinct estimates and interval methods side by side.

**Table 2:** Sensitivity of the marker association to the comparison used Each row is one estimate of the association between an unavailable-information marker in the first simulator feedback and reaching the fifth assistant response, in percentage points, with its 95% confidence interval (CI) and the method used to obtain the interval. The equal-weighted summary and the regression coefficients weight runs differently and should not be compared directly. The two full-corpus regression models use the same 5,719 runs from 954 source-report clusters and the same common-slope specification, so comparing them shows the effect of adding report indicators. The blood, heart and circulation subset contains 111 report clusters. CR1 denotes the finite-sample-adjusted cluster-robust sandwich variance with normal quantiles. The blood, heart and circulation subset row is exploratory and is not interpretable as an effect estimate: exposure cells were empty in some bootstrap resamples, and one model had only three marker-negative runs. None of these comparisons randomizes information availability.

Comparison	Eligible runs	Difference or coefficient, percentage points	95% CI lower limit	95% CI upper limit	Interval method
Equal-weighted mean of seven model-specific differences	5719	6.37	4.16	8.54	Source-report percentile bootstrap
Common-slope linear probability model with model effects	5719	5.61	3.41	7.82	Source-report CR1 cluster sandwich
Common-slope linear probability model with report and model effects	5719	1.05	-1.64	3.74	Source-report CR1 cluster sandwich
Report and model effects, blood, heart and circulation subset (exploratory; not interpretable as an effect estimate)	677	2.91	-6.88	12.69	Source-report CR1 cluster sandwich

The report-adjusted coefficient rests on part of the data. Of the 954 source reports with eligible runs, 642 (3,935 runs) contributed both marker-positive and marker-negative runs; 306 contributed only marker-positive runs and six only marker-negative runs, and these 312 reports inform the model effects but not the within-report contrast. Both marker status and fifth-response contact varied within 369 reports. Adding the number of requested items and the six request-category indicators of the first request to the report-and-model regression changed the coefficient from 1.05 to 1.21 percentage points (95% CI -1.67 to 4.08); with model effects only and the same request covariates, it was 4.37 points (1.99 to 6.75). The attenuation after report adjustment therefore did not depend on these observable differences in request composition, although request content was characterized only lexically.

Separating the marker groups was more informative. Relative to marker-negative first feedback, mixed feedback was associated with a 4.52-point higher fifth-response proportion with model effects (95% CI 2.34 to 6.70) and 0.72 points with report and model effects (-1.97 to 3.42). The 173 marker-only feedback showed a larger association: 29.86 points with model effects (22.74 to 36.99) and 13.04 points with report and model effects (3.69 to 22.40). Of these runs, 40.5% reached the fifth response, compared with 17.7% after mixed and 13.1% after marker-negative feedback. This exploratory contrast rests on 173 runs defined by a lexical rule, but it indicates that the weak overall within-report association combines a small group in which nothing else was returned with a large group in which the marker accompanied other returned text.

The wording sensitivity changed 25 first-feedback classifications, and the equal-model-weighted difference was 6.39 percentage points, close to the exact-marker result. This check addresses a narrow wording variation; it is not a validation of semantic information availability. Restricting to the 948 reports with all seven released model outputs yielded a difference of 6.32 percentage points (95% report-cluster bootstrap interval 4.11 to 8.45), with all 5,000 resamples estimable.

Cardiovascular and heart failure applicability

The exact blood, heart, and circulation rule identified 111 reports and 775 released runs from 777 expected cells (Table 3). The two absent outputs were MedGemma for PMC11549668 and DiagnoseGPT for PMC11552470. Of the released runs, 98 had no feedback, and 677 entered the first-feedback analysis. The marker was present in 572 first-feedback runs, and 154 runs reached the fifth response.

**Table 3:** Data coverage and heart-failure term screening in the MedR-Bench diagnosis release Each row gives, for one analysis set or searched text field, the number of source case reports and, where the set was analyzed, the expected report-model runs (seven per report), the runs actually released, and the runs that entered the first-feedback analysis. Heart-failure rows come from a case-insensitive word-boundary search for heart failure, cardiac failure, heart failure with reduced ejection fraction (HFrEF), heart failure with preserved ejection fraction (HFpEF), or heart failure with mildly reduced ejection fraction (HFmrEF); the text-screened rows overlap and are not adjudicated human heart-failure cohorts. Only four summary-positive reports belong to the blood, heart and circulation metadata subset. Source reports are not verified as unique patients. The 496 treatment reports were inspected separately and are not added to these counts.

Analysis set or text field	Source reports	Expected runs	Released runs	First-feedback eligible runs
All diagnosis reports	957	6699	6690	5719
Blood, heart and circulation metadata subset	111	777	775	677
Heart-failure term in case summary	17	119	119	104
Heart-failure term in generated diagnosis results	2	Not analyzed as a cohort	Not analyzed as a cohort	Not analyzed as a cohort
Heart-failure term in generated final diagnosis	5	Not analyzed as a cohort	Not analyzed as a cohort	Not analyzed as a cohort
Heart-failure term in any source field	60	Not analyzed as a cohort	Not analyzed as a cohort	Not analyzed as a cohort

The equal-model-weighted contrast in this subset was 11.18 percentage points. This estimate was fragile: QwQ had only three marker-negative eligible runs, and exposure cells became empty in 252 of 5,000 report resamples. We, therefore, report the subset as an applicability analysis and do not use a conventional pooled CI to support a cardiovascular-specific effect claim. With report and model effects, the subset coefficient was 2.91 percentage points (95% cluster-robust interval -6.88 to 12.69). These subset contrasts are hypothesis-generating at most.

Heart-failure term screens identified two reports in the generated diagnosis-results field, five in the generated final-diagnosis field, 17 in case summaries, and 60 when any source field was searched (Table 3). These are overlapping sets with different meanings, not alternative estimates of a confirmed disease prevalence. Only four of the 17 summary-positive reports belonged to the blood, heart and circulation metadata subset, so the lexical screen is a parallel screen of all diagnosis reports rather than a nested cardiovascular cohort. The 17 summary-positive reports yielded 119 released runs, including 104 eligible runs; one model had no marker-negative eligible run, so a seven-model heart-failure contrast was not estimable. The treatment inventory contained 11 summary-positive reports and 27 with a mention in any field, but no treatment-output comparison was available.

Targeted source checks further limited interpretation. Of the two diagnosis-results matches, PMC11462620 describes a cat with cardiac lymphoma and congestive heart failure [11], while PMC11402539 concerns postmortem imaging after sudden death [12]. These matches do not establish a human heart-failure cohort. A broader text-based source-context audit identified at least eight explicitly nonhuman diagnosis reports, including one report describing five sheep. Excluding these eight reports left 949 source reports and 5,685 eligible runs; the equal-model difference was 6.36 percentage points (point sensitivity only). This check does not certify that every remaining report represents a living human clinical encounter.

Lexical audit findings

An entire information-request block was repeated verbatim within 430 released runs (6.4%); after normalization, 446 runs (6.7%) contained a repeated block. Only 2,301 runs had at least two noncanonical request sections and therefore an opportunity for a repeated-block event under this rule; the corresponding proportions among those runs were 18.7% and 19.4%. The audit also found 16 runs in which at least one assistant response lacked a literal required heading. These observations characterize the release and parser behavior, not the clinical necessity of an examination or correctness of a conclusion.

The permissive substring stop rule matched 30 terminal sections that were not canonical stop phrases. Thirteen terminal sections instead used a short "none" or "none required" synonym. These results show why extraction decisions must be exposed in a trajectory audit. They do not establish that every ambiguous section contained an unresolved clinical request.

Paired protocol extension

The additional files contained 6,680 one-information-round runs and 6,699 oracle outputs. Together with the 6,690 free-turn runs, they formed an inventory of 20,069 condition-specific outputs on the same 957 source reports. All three conditions were available for 6,672 report-model cells, and 931 reports had a complete seven-model, three-condition grid. Across the 6,672 one-round/free-turn pairs, the extracted case section and initial system message were identical, but the complete initial user message and first assistant answer were never identical. Among 5,701 pairs with first feedback in both conditions, only two extracted feedback bodies matched exactly. The oracle file supplied model answers without an independent correctness score.

The exact marker appeared in 4,778 one-round feedback (83.8%) and 4,575 free-turn feedback (80.2%). There were 4,022 marker-positive pairs under both conditions, 756 one-round-only, 553 free-turn-only, and 370 negative under both (Table 4). Thus, 1,309 pairs (23.0%) changed marker state. The equal-model-weighted free-turn-minus-one-round difference was -2.90 percentage points (95% report-cluster bootstrap interval -4.26 to -1.52). This average was strongly model-dependent: Baichuan-M1 differed by -20.28 points, while the arithmetic mean for the other six labels was approximately zero. The marker was therefore not an invariant property of the shared source-case text. These comparisons do not identify an isolated effect of the turn budget, and a lower marker rate does not establish better clinical information acquisition.

**Table 4:** Paired state changes across interaction protocols in two public benchmark releases Each row cross-classifies paired observations of the same source material under two protocols of one benchmark: the number in which the stated outcome occurred under both protocols, under the reference protocol only, under the comparison protocol only, and under neither. MedR-Bench pairs are report-model cells with first simulator feedback under both the one-information-round and the free-turn release (1,309 of 5,701, 23.0%, changed state). CARE-Bench pairs are case-model combinations from 62 cases and 11 model labels, using the developers' mapped predictions. Prompts, generated responses and feedback differed between protocols, so neither row isolates an effect of the protocol. CARE-Bench counts describe agreement with a benchmark reference, not clinical correctness or harm.

Paired comparison	Outcome state	Both conditions, n	Reference condition only, n	Comparison condition only, n	Neither condition, n	Total pairs, n
MedR-Bench, reference one information round, comparison free turn	Unavailable-information marker in first feedback	4022	756	553	370	5701
CARE-Bench Public Test 1, reference unprompted, comparison prompted	Agreement with the action reference at every prefix of a case	106	44	132	400	682

The first requests also differed between releases. The number of requested items was the same in 1,926 of the 5,701 pairs (33.8%), and the set of lexical request categories was the same in 2,277 (39.9%); on average, the free-turn first request listed 0.48 fewer items. In the 966 pairs whose first requests matched on both item count and category set, marker status still differed in 197 (20.4%), compared with 1,112 of 4,735 (23.5%) in the remaining pairs. Request differences therefore explain only part of the discordance, and these comparisons remain descriptive differences between released protocol conditions.

Separate triage-benchmark reanalysis

The CARE-Bench mapped-output grid was complete at 2,970 predictions: 62 cases, 135 prefixes, 11 model labels, and two protocols. Actual-model, provider, mapper-model, and reference-label fields agreed across all 1,485 paired prefix records. We reproduced the previously reported mean trajectory exact match of 22.0% unprompted and 34.9% prompted [10]. The added paired decomposition comprised 106 case-model combinations agreeing at every prefix under both protocols, 132 under prompted only, 44 under unprompted only, and 400 under neither (Table 4). The net difference was 12.90 percentage points (95% whole-case bootstrap interval 7.48 to 18.33); gains and losses represented 19.4% and 6.5% of the 682 case-model pairs, respectively. Thirty of the 62 cases had a loss of complete reference agreement for at least one model, and 21 had gains for some models and losses for others. These are changes in benchmark-reference agreement, not observed clinical harm.

## Discussion

The central finding is that a seemingly clear feedback-behavior association changes when the comparison accounts for source reports. The equal-model summary was positive, and so was a model-only regression. Adding report effects reduced the common-slope estimate from 5.61 to 1.05 percentage points, with an interval spanning zero. Because these regressions use the same sample and coefficient definition, the comparison is more informative about case composition than directly contrasting the regression with the differently weighted model average. Stated plainly, the positive equal-weighted difference of 6.37 points is largely a product of case composition, not evidence that the marker predicts fifth-response contact within a case: the report-adjusted coefficient was small, it stayed small after adjustment for request composition, and one model label carried much of the unadjusted summary. The analysis still does not identify a causal effect. The exposure also needs a caveat at the outset: in 96.2% of marker-positive first feedback, the simulator returned other text for at least one requested item, so the marker indexes a visible simulator string and not a measured quantity of missing clinical evidence. Its construct validity is, therefore, limited, and every estimate in this paper should be read with that limitation.

The saved prompts supply a second constraint: every fifth response followed a mandatory-final-answer instruction. Treating all final responses as the same stopping decision would therefore combine different protocol states. The data show contact with that constraint; they cannot show how long those conversations would have continued without it. For this reason, we describe the outcome as fifth-response contact and keep it distinct from earlier endings throughout; neither should be read as a measure of inappropriate continuation, inefficient reasoning, or a model's own stopping decision.

The marker requires equally careful interpretation. Most marker-positive first feedback contained additional text. The item-level decomposition showed that 96.2% of marker-positive first feedback also returned other text for at least one requested item, so the binary marker usually indicates partial rather than complete unavailability. The small marker-only group behaved differently from the mixed group, which suggests that the extent of unavailability, rather than the presence of the sentence, may be the more relevant quantity; a clinically adjudicated sample would be needed to confirm this. The result is consequently about a visible simulator response, not a measured quantity of missing clinical evidence. That narrower construct is reproducible from the release and can be checked without asking an automated judge to infer clinical appropriateness.

This analysis extends the original MedR-Bench evaluation with a first-feedback observation point, report-clustered contrasts, and explicit verification of the protocol-control prompt. The original investigators already described repeated queries and difficulties with stopping [1]. We do not present those phenomena as new discoveries. The additional contribution is to quantify how one observable feedback signal relates to later protocol contact while preserving the cases that never reach the observation point and the outputs that were not released. These distinctions also complement broader work on evidence grounding and benchmark integrity [4,6].

Several mechanisms could produce the association. Difficult cases may elicit both less answerable requests and more interaction. Models may request different examinations for the same report. The simulator may respond differently to wording or to a bundled list of requests. A model that usually answers immediately enters our analysis only on an unusual subset of its cases: entry ranged from 41.2% of released runs for DiagnoseGPT to 98.0% for Baichuan-M1, so the first-feedback sample is itself strongly selected, and the model-specific contrasts refer to different case mixes. The first-feedback design avoids using future opportunities to define exposure, but it does not eliminate any of these mechanisms. More rounds should therefore not be called inefficient, and earlier endings should not be called successful.

For cardiovascular research, the applicability analysis provides a useful constraint on study design. The available category supports a broad blood and circulation subset, but the evidence for a heart-failure-specific study is much thinner and depends strongly on the searched field. Moving directly from these data to a heart-failure clinical-validation claim would change the population and outcome without supplying the required evidence. The feline and postmortem examples make this distinction concrete: clinical terms alone do not establish species, care setting, or a patient denominator. A future heart-failure study would need an independently defined clinical cohort, interpretable information opportunities, and a reference that distinguishes absent documentation from inappropriate care. The present data can inform its extraction design but cannot stand in for that validation.

The protocol extensions sharpen this interpretation without supplying external validation of a common endpoint. They were included because the measurement question of this paper, whether a protocol-conditioned trajectory summary can be read as a model property, arises in both benchmarks, even though contact with an interaction limit and agreement with an action reference are different endpoints that we do not combine. MedR-Bench preserved the source-case section across conditions, but full prompts, generated requests, and feedback differed. Even among pairs whose first requests matched on item count and category, one in five changed marker status, so the discordance is a descriptive difference between released conditions and not an effect of the turn budget. CARE-Bench supplied categorical action references and mapped predictions, but no raw responses for independent mapping review. Its average trajectory exact-match improvement was already published; our increment is the paired gain and loss decomposition and its case-level uncertainty. An aggregate improvement can coexist with losses in individual case-model combinations. Neither setting supports treating a protocol-conditioned summary as a model characteristic that is constant across information and instruction settings.

Limitations

The primary analysis is a secondary analysis of one publicly released benchmark artifact; the triage extension addresses a different task and reference system. Published case reports are selected educational records, often chosen because they are unusual, and they do not represent routine diagnostic encounters. Source-report identifiers do not prove patient independence, and prior model exposure to the publications cannot be excluded. The models and simulator were not rerun, so stochastic variability and contemporary versions were not evaluated.

The exact-sentence rule misses semantically similar replies and does not validate the simulator against the original ancillary results. Neither marker-negative nor mixed-content feedback guarantees useful information. The first-feedback sample conditions on the model requesting additional information, and models differ substantially in that selection (41.2% to 98.0% of released runs). This is a strong selection effect: every contrast in this paper applies only to the selected runs. The item-level decomposition and the request covariates are lexical, and they were added after peer review. Bootstrap intervals account for reuse of the same source report across models, but do not make the marker groups exchangeable or supply external clinical validity. All analyses were developed after some data inspection, and the small cardiovascular and heart-failure subsets support limited inference.

The CARE-Bench extension inherits its developers' case construction, action references, and response mapping. Its public predictions cover 62 test cases rather than all 439 public cases. Case-complete agreement is a conjunction over one to three evaluated prefixes and is sensitive to that opportunity count. Resampling cases does not measure stochastic variation from repeated model generation. Finally, no independent clinical judgments were collected, so we cannot estimate diagnostic accuracy, clinical error severity, patient benefit, or the value of any cardiovascular clinical scoring system from these results.

## Conclusions

An explicit unavailable-information text marker was common in these released conversations, and in most cases it accompanied other returned text. Its positive association with fifth-response contact across models was largely explained by case composition: within source reports, the association was close to null and did not change materially after adjustment for request composition. Every fifth response also followed a mandatory-final-answer instruction, so fifth-response contact is a protocol state and not a stopping decision. Evaluation reports should retain case composition, simulator feedback, and protocol-control prompts when interpreting diagnostic trajectories. The current release does not establish a clinical effect of missing information or heart-failure-specific model performance.
